# Prevalence of HIV and Syphilis Infection among High School and College Student MSM in China: A Systematic Review and Meta-Analysis

**DOI:** 10.1371/journal.pone.0069137

**Published:** 2013-07-16

**Authors:** Yongze Li, Junjie Xu, Kathleen H. Reilly, Jianming Zhang, Hongyi Wei, Yongjun Jiang, Wenqing Geng, Weiming Tang, Hong Shang

**Affiliations:** 1 Key Laboratory of AIDS Immunology of Ministry of Health, Department of Laboratory Medicine, No 1 Hospital of China Medical University, Shenyang, Liaoning, China; 2 National Center for AIDS/STD Control and Prevention, Chinese Center for Disease Control and Prevention, Beijing, China; 3 Department of Clinical Epidemiology and Evidence-based Medicine, No 1 Hospital of China Medical University, Shenyang, Liaoning, China; 4 Department of Epidemiology, Fielding School of Public Health, University of California Los Angeles, Los Angeles, California, United States of America; University of Southern California Keck School of Medicine, United States of America

## Abstract

**Objectives:**

The aim of this study is to systematically review the published studies and summarize the estimates of HIV and syphilis prevalences among high school and college student MSM in China in order to provide information for conducting targeted interventions.

**Methods:**

Pubmed, Chinese National Knowledge Infrastructure, Wanfang and Google Scholar databases were searched in January 2013 to identify relevant articles. Data of eligible citations were extracted by two reviewers. All analyses were performed using Comprehensive Meta-Analysis V2.0 and SPSS V17.0.

**Results:**

Twenty-four eligible studies (6 in English and 18 in Chinese), published between 2006 and 2012, with a total of 3083 student MSM participants, were included. The meta-analyses showed that the prevalences of HIV and syphilis among studied student MSM were 4.4% (95% CI: 3.0%, 6.4%) and 5.7% (95% CI: 4.8%, 6.7%), respectively. HIV prevalence increased over the study period (3.0% in 2003–2006; 4.5% in 2007–2008, and 6.8% in 2009–2010, trend test chi-square = 11.3, p = 0.001).

**Conclusions:**

Student MSM have become high-risk populations for HIV infection in China. The high prevalence of syphilis and the increasing HIV prevalence trend indicate the potential for a more severe HIV epidemic. Comprehensive intervention strategies that address condom promotion, syphilis detection and treatment, and health education need to be tailored to this vulnerable population to prevent HIV and syphilis infections.

## Introduction

According to a report from the Chinese Ministry of Health, it estimated that there were approximately 780,000 people living with human immunodeficiency virus (HIV)/acquired immune deficiency syndrome (AIDS) in 2011 in China [1]. Sexual transmission is the primary mode of transmission and cases attributed to sexual transmission continue to increase [1]. Among the 7.8 million people living with HIV (PLHIV), 46.5% were infected by heterosexual transmission and 17.4% were infected by homosexual transmission [1]. Homosexual transmission has increased since surveillance of HIV among this group began a decade ago. In 2011, 29.4% of the estimated 48,000 new HIV infection were infected through homosexual transmission [1].

AIDS is currently one of the leading causes of death among 15- to 24-year old adolescents and young adults worldwide [2]. Many in this age group are high school and college students and China has the largest number of students in the world [3]. According to a report from the Chinese Ministry of Education, there were an estimated 46.8 million high school students and 31.1 million college students in China by the end of 2010, approximately half of whom are male [4].

Chinese students were not previously considered a population at high-risk for HIV infection [5]. However, among nationally reported HIV/AIDS cases, the proportion of HIV among students increased from 0.96% to 1.64% in 2006 and 2011, respectively. During this time period (2006–2011), among self-reported students, the proportion of HIV cases among 20–24 year old group increased from 20.3% to 49.0% and the proportion of homosexually transmitted cases increased from 8.0% to 55.5% [1]. However, only a few studies with small samples sizes have been conducted to analyze the prevalence of HIV infection among Chinese student MSM [3], [6].

Understanding the prevalence of HIV and other sexually transmitted infections (STIs) among Chinese student MSM is important in order to design proper interventions for this population to curb the HIV epidemic [7]–[9]. The objective of this study is to summarize the prevalence of HIV and syphilis among high school and college student MSM in China by conducting a meta-analysis of published studies. Trends in HIV prevalence were also assessed.

## Methods

### Literature Search

Pubmed, Chinese National Knowledge Infrastructure, Wanfang and Google Scholar databases were searched up to January 5, 2013 to identify relevant articles. The keywords and medical subject headings “HIV,” “AIDS,” “MSM,” “gay,” “homosexual,” “student,” and “China” were used to screen for potentially relevant studies.

### Inclusion and Exclusion Criteria

Studies were eligible for inclusion in the review if they met the following criteria: (1) published in Chinese or English language; (2) study participants were Chinese high school or college male students; (3) study participants reported homosexual behavior (recall window ≤12 months) and presented HIV and/or syphilis prevalence; and (4) HIV and/or syphilis diagnosis was conducted in a laboratory with standard serologic testing methods. Studies were excluded if they met the following criteria: (1) not an original study, such as a review paper; (2) local/government reports and conference abstracts; (3) study participants were employed or no longer students; and (4) if the same study data were published in both English and Chinese, articles published in Chinese were excluded from the review.

### Data Extraction and Study Quality Assessment

Data from eligible studies were extracted independently by two reviewers (Y Z Li and H Y Wei). The following data were extracted from all studies: first author and year of publication; study site and period; sampling methods; sample size; and results of HIV and syphilis tests (if available). Disagreements between the two reviewers during data extraction were reconciled by a third investigator (J J Xu).

The quality assessment checklist for observational studies (QATSO score) was selected to evaluate the quality of the included studies [10]. Items were scored as 1, 0, NA, which corresponded to “yes”, “no”, or “not applicable”, respectively. The total score was divided by the total number of all applicable items. Values between 0% and 33%, 33% and 66% and 67% and 100% represent “bad”, “satisfactory” and “good quality,” respectively.

### Statistical Analysis

Meta-analysis of the prevalence of HIV and syphilis infection was carried out using the Comprehensive Meta-Analysis software (V2.0, Biostat, Englewood, NJ). Stratified analyses were performed by study locations, study year, sample size, and sampling methods. Changes of crude HIV prevalence among studied student MSM over the study period and the difference between the pooled prevalence and national prevalence of HIV and syphilis were analyzed using the Chi-square test for linear trend using SPSS (V17.0, Chicago, IL, USA). Heterogeneity between studies was examined using the Q test (p<0.10 was considered indicative of statistically significant heterogeneity) and the I^2^ statistic (values of 25%, 50% and 75% are considered to represent low, medium and high heterogeneity, respectively) [11]. Random effects models were used in the meta-analysis to take into account the possibility of heterogeneity. The Begg rank correlation method was used to assess the potential for publication bias (p<0.05 was considered the indicative of statistically significant publication bias). Analyses were conducted separately for both HIV and syphilis.

## Results

### Study Selection for Meta-Analysis

In total, 1358 citations were identified, of which 48 potentially relevant articles were selected for further screening, and eventually 24 articles (6 published in English and 18 in Chinese), with 3083 student MSM participants met the inclusion criteria (Figure S1). Table 1 [3], [6], [12]–[33] provides a descriptive summary of these studies. In most studies, blood samples were tested for HIV and syphilis. For all of the studies, the presence of HIV-1 antibody was initially tested by enzyme-linked immunosorbent assay (ELISA), and positive/indeterminate tests were confirmed by HIV-1/2 Western Blot (WB) assay. For most of the studies that tested for syphilis, participants with serum positive for both Treponema pallidum particle assay (TPPA) and rapid plasma regain (RPR) were determined to be currently infected with syphilis. All the included studies met the criteria of “good quality” (values between 67% and 100%) in study quality assessment.

**Table 1:** Prevalence of HIV infection among student MSM in China.

First author, published year	Study design				Prevalence % (n)	
	Location	Sampling method	Study year	Sample size	HIV infection	Syphilis
Xu JJ, 2011	Liaoning	MSM NGO invited	2008–2009	436	3.0% (13)	5.0% (22)
Zhang L, 2012	Chongqing	RDS	2009	183	5.5% (10)	4.4% (8)
He Q, 2006	Guangzhou	Media and MSM peer	2003	14	0% (0)	Not Reported
Zhu JL, 2007	Hefei	RDS,MSM peer,media	2005	121	1.7% (2)	7.4% (9)
Zheng JD, 2008	Beijing	Media	2007	27	0% (0)	7.4% (2)
Zheng JD, 2011	Beijing	The Internet, NGO	2007	157	2.5% (4)	Not Reported
Wei S, 2011	4 cities^a^	Snowball sampling	2008	205	2.9% (6)	4.9% (10)
Ruan YH, 2007	Beijing	the Internet, community outreach	2005	108	1.8% (2)	4.6% (5)
Zhang XX, 2007	Beijing	VCT	2005–2006	130	1.5% (2)	Not Reported
Xu ZH, 2009	Tieling	MSM venues	2007	20	0% (0)	Not Reported
Zheng JD, 2008^b^	Beijing	Snowball sampling and the Internet	2007	175	2.3% (4)	7.4% (13)
He QY, 2011	Chengdu	Snowball sampling	2008	169	8.9% (15)	5.3% (9)
Feng LG, 2010	Chongqing	Snowball sampling	2009	190	11.1% (15)	5.8% (11)
Ma AB, 2007	Yunnan	VCT	2005	9	1.3%(1)	Not reported
Guo XJ, 2011	Chongqing	Snowball sampling	2006	211	4.7%(10)	7.1% (15)
Zhou JB, 2012	Changzhou	MSM venues, the Internet,MSM peer	2009	26	26.9%(7)	15.4% (4)
Fu LJ, 2007	Shaoxing	Snowball sampling	2006	12	0% (0)	Not Reported
Tang L, 2006	Wuhan	VCT	2006	43	25.6% (11)	Not Reported
Chen SH, 2007	Nanning	VCT	2006	45	0% (0)	Not Reported
Wang LX, 2010	9 cities^c^	Snowball sampling	2005–2006	324	0.6%(2)	Not Reported
Du GY, 2011	Liaocheng	Snowballing, media, MSM venues	2010	89	1.1% (1)	2.2% (2)
Zhou ZH, 2010	Beijing	VCT	2008	60	3.3% (2)	Not Reported
Zhou C, 2010	Chongqing	Snowball sampling	2008	253	9.1%(23)	3.6%(9)
Su YZ, 2011	Kunming	Snowball sampling	2006	76	3.95% (3)	Not Reported

a 4 cities include Harbin, Beijing, Zhengzhou, Chengdu;

b This study is different from Zheng JD, 2008;

c 9 cities include Harbin, Shenyang, Xi’an, Zhengzhou, Nanjing, Shanghai, Wuhan, Chongqing, Chengdu.

### Pooled Prevalence of HIV and Syphilis Infection Among Student MSM

Figure S2 shows the summarized national estimate of HIV prevalence among student MSM from 2003–2010 (4.4%; 95% CI: 3.0%, 6.4%) with study prevalences ranging from 1.1% (95% CI: 0.1%, 15.1%) to 26.9% (95% CI: 13.4%, 46.7%). No significant publication bias was observed (p = 0.82). However, there was substantial heterogeneity between studies (p for Q test, p<0.01; I^2^ = 75.35). As shown in Figure S3, a high prevalence of syphilis seropositivity was observed in the student MSM population among included studies with a summarized estimate of 5.7% (95% CI: 4.8%, 6.7%), with study syphilis prevalences ranging from 2.2% (95% CI: 0.6%, 8.5%) to 15.4% (95% CI: 5.9%, 34.5%). There was no heterogeneity between studies (p = 0.44; I^2^ = 0.70). No publication bias was observed as assessed by the Begg rank correlation analysis (p = 0.86).

### Stratified Analyses across Studies and the Trend Test of HIV Prevalence over Time

Stratified analyses were conducted for sampling methods, sample size, study locations, and study year (Table 2). Heterogeneity between studies was partly reduced in subgroup analyses. The subgroup of studies with larger sample size (n>200) had a lower prevalence of HIV (4.9%; 95%CI: 3.7%, 6.3%). As seen in Figure S4, the high prevalence of HIV among student MSM were mostly found in Sichuan Province and Chongqing. The summary prevalence in the southwest was the highest (7.3%; 95%CI: 5.8%, 9.1%), followed by eastern China (6.8%; 95%CI: 2.4%, 68.6%), central China (6.4%; 95%CI: 0.7%, 41.0%), northeast (3.0%; 95%CI: 1.7%, 5.0%), northern China (2.3%; 95%CI: 1.4%, 3.7%), and southern China (1.9%; 95%CI: 0.2%, 12.3%). From 2003 to 2010, there were 24 reported HIV prevalence estimates among student MSM in China. The pooled estimate across all studies suggests that HIV prevalence among this population has increased substantially: it was 3.0% (33/1093) in 2003–2006, 4.5% (67/1502) in 2007–2008, and 6.8% (33/488) in 2009–2010. HIV prevalence experienced a significant sustained increases over these three time periods (trend test chi-square value = 11.3, p = 0.001). The difference between the pooled prevalences of HIV (4.4% vs. 6.3%, chi-square value = 19.7, p<0.001) and syphilis (5.7% vs. 10.0%, chi-square value = 49.2, p<0.001) were both significantly lower than the national prevalence of HIV and syphilis among MSM.

**Table 2:** Stratified meta-analyses of HIV prevalence among student MSM of China.

Subgroups	Prevalence%(95%CI)	No of studies	Heterogeneity	
			I^2^%	p Value
Sample size				
> 200	4.9% (3.7%–6.3%)	5	82.72	<0.05
≤200	6.9% (5.5%–8.5%)	19	72.82	<0.05
Study year				
2003–2006	3.4% (1.4%–7.7%)	11	78.89	<0.05
2007–2008	4.1% (2.5%–6.7%)	9	67.54	<0.05
2009–2010	7.8% (3.2%–1.8%)	4	81.57	<0.05
Study location				
Central China	6.4% (0.7%–41.0%)	3	87.16	<0.05
East China	6.8% (2.4%–68.6%)	2	90.03	<0.05
National	1.5% (0.3%–6.6%)	2	72.96	>0.05
North China	2.3% (1.4%–3.7%)	6	–	>0.05
Northeast	3.0% (1.7%–5.0%)	2	–	>0.05
South China	1.9% (0.2%–12.3%)	2	–	>0.05
Southwest	7.3% (5.8%–9.1%)	7	3.70	>0.05
Sampling method				
MSM NGO & Media	4.6% (2.0%–10.1%)	12	81.53	<0.05
Snowball sampling & RDS	5.3% (3.5%–7.8%)	9	65.06	<0.05
Mixed recruitment methods	1.9% (0.9%–3.9%)	3	–	>0.05

## Discussion

This review assessed the prevalences of HIV and syphilis infections, and trend of HIV over time among Chinese high school and college student MSM. The summarized estimates of prevalence of HIV (4.4%; 95% CI: 3.0%, 6.4%) and syphilis (5.7%; 95% CI: 4.8%, 6.7%) infection were both somewhat high. The increasing trend of HIV prevalence among Chinese student MSM suggests that the epidemic could increase in the absence of effective public health strategies targeting this population. Understanding student MSM is important in order to design proper interventions to curb China’s HIV epidemic.

The prevalence of HIV infection among student MSM is over seventy times higher than the Chinese general population (4.4% vs. 0.06%) [1]. This prevalence is also higher than that reported among student MSM in Spain (1.6%) and Seattle, USA (2.2%), but much lower than the prevalence of HIV (49%) reported among young non-Hispanic black college student MSM in North Carolina, USA [34]–[36]. Our results of the prevalences of HIV and syphilis were markedly lower than that reported among all MSM nationally (HIV 6.3% and syphilis 10.0%) [1]. The HIV prevalence in the current study is somewhat lower than that found in another study of non-student MSM (20.9%) in China [6]. It is possible that student MSM in our research were younger, and therefore had shorter durations of potential exposure. However, the HIV prevalence of 4.4% is still quite high, and close to the level considered as HIV concentrated epidemics (5.0%) [37]. The results of the current study indicate that the HIV epidemic among student MSM in China is far more serious than previously thought.

Regions varied in their prevalence of HIV infection. The HIV infection rate of student MSM in southwest China was the highest (7.3%). A possible explanation for the high prevalence in the southwest area is that this area has a more developed economy and a dense population, but is located along a major transportation route and the neighboring provinces are severely affected by HIV via injection drug use [38]–[40]. The population mobility in this region is high and a part of the subculture [41]. Moreover, the HIV prevalence among all MSM in the southwest is remarkably high in comparison with other regions in China [42].

The high prevalence of syphilis (5.7%) among student MSM may indicate a high prevalence of unprotected sex and suggests a potential risk for the rapid spread of HIV among student MSM, as syphilis is more infectious than HIV [43]. Syphilis may also act as a cofactor for HIV seroconversion [17], [44], [45] and those who have syphilis infections maybe more likely to contract HIV [46]. On the other hand, because of the behavioral and biological links between syphilis and HIV, the two infections could be simultaneously addressed among student MSM to reduce the incidence of both.

The varieties of subpopulations of Chinese MSM have different demographic and sexual risk “profiles” that suggest high potential for bridging infection across geographies, generations and genders [47]. Ma and Cong found that in Zhejiang province, 5.0% of male university students had had sex with men and 2.6% had engaged in bisexual behaviors [48]. Bisexual student MSM may play a bridging role in the spread of HIV and other sexual transmitted diseases from high-risk populations to the general population. Yun et al. have found high HIV (5.4%) and syphilis (11.4%) prevalences among MSM engaging in bisexual behavior and HIV prevalence among MSM engaging in sex with both men and women was significantly higher than in those who only have sex with men [11]. Most included studies did not provided detailed data on HIV infection and syphilis-related risk factors, which makes it difficult to ascertain the correlation between HIV and syphilis infection. Additional large-scale epidemiological investigations among student MSM are required to better understand their population size, status of the epidemic, and their demographic characteristics, which can aid in the design of comprehensive and effective interventions for student MSM.

The Chinese government should take action, both the Ministries of Education and the Ministry of Health should allocate greater resources for HIV prevention and control among college students in China, which as the results of this study indicate, is a vulnerable population for HIV infection. Sexually active college student MSM are likely to migrate to other parts of the country after graduation, which could lead to spread of the virus and create challenges towards HIV prevention and control. Morever, the alarming spread of HIV among MSM is fuelled by an ongoing, persistent stigma against homosexuality in China [49]. The result is student MSM hiding their MSM population from the general student population, thereby increasing the difficulty of conducting interventions among this population. Without adequate interventions, effective implementation, and strategies targeted towards this population, the epidemic will continue to spread in China. How China handles the issue may largely depend on reducing risk factors for student MSM; promoting HIV/syphilis screening, diagnosis, referral and treatment among students MSM; offering free condoms in schools; removing the stigma of homosexuality and fighting discrimination against homosexuals; promoting health education aimed at this group [42]; and encouraging widespread family and school-based campaigns through China’s mainstream media [3].

To our knowledge, this is the first meta-analysis of the prevalence of HIV among student MSM in China. This study is, however, subject to several limitations. Of the 24 included studies, sampling methods, study year, and study locations varied; the sample size ranged from 9 to 436 participants. The results of these studies were heterogeneous. Differences between sampling methods, study year, sample size and study locations need to be taken into account and may explain some of the inconsistencies observed between studies. There are less longitudinal studies of HIV infection among student MSM and more cross-sectional studies, which cannot describe changes in sexual behavior over time. Furthermore, some of the included studies were sampled through RDS and snowball sampling rather than population-based, which makes the results more prone to selection bias [43]. Also, the studies included in this meta-analysis were concentrated in the southern, southwest, northern, and central regions of China and there was a lack of data from the northwest. Therefore, the results of this meta-analysis do not represent all regions of China. Small sample sizes also restricted the power of subgroup analyses. Another limitation is that publication bias cannot be excluded, although no indication of major publication bias was significant in the meta-analysis. In the stratified analyses, heterogeneity between studies was reduced, suggesting that the varied data collection methods, sample size, study time and study locations may account for the inconsistency among studies. A higher prevalence of HIV infection was found among studies with sample sizes ≤200 compared with sample sizes >200. As our results suggest, the prevalence may be overestimated by the studies with smaller sample sizes. In addition, regional variations should be considered, as the prevalence of HIV in southwest China is remarkably high in comparison with the other study sites.

Student MSM form a high-risk population for HIV infection and syphilis in China. Long-term, comprehensive and effective prevention interventions should be considered for this population. Further large-scale epidemiological investigations, with standard sampling methods and adequate power, should be conducted to gain a more precise estimate of the status of the HIV and other sexually transmitted infection epidemics among student MSM in China.

## Supporting Information

Figure S1 **Flow diagram of the study selection process.** As shown, our initial searches yielded 1,358 citations from Pubmed, Chinese National Knowledge Infrastructure, Wanfang and Google Scholar databases. After screening titles and abstracts, 48 studies were considered potentially eligible and retrieved in full text. Of these, 24 studies were subsequently excluded because they did not satisfy the inclusion criteria. Thus, 24 fully eligible studies were identified.
(TIF)

Figure S2 **Meta-analysis of the HIV prevalence among student MSM in China.** Figure S2 shows unadjusted HIV prevalence estimates (boxes) with 95% confidence limits (bars) for each study selected; pooled prevalence estimates are represented as diamonds in this plot. ^*^ This study is different from Zheng JD, 2008.
(TIF)

Figure S3 **Meta-analysis of syphilis prevalence among student MSM in China.** Figure S3 shows unadjusted syphilis prevalence estimates (boxes) with 95% confidence limits (bars) for each study selected; pooled prevalence estimates are represented as diamonds in this plot.^ *^ This study is different from Zheng JD, 2008.
(TIF)

Figure S4 **The regional distribution of pooled prevalence of HIV among student MSM in China.**
(TIF)

Checklist S1 **PRISMA Checklist of this meta-analysis.**
(DOC)
