# Supplementary figures and images for: Prevalence of HIV and Syphilis Infection among High School and College Student MSM in China: A Systematic Review and Meta-Analysis
Source: PLoS One. 2013 Jul 16;8(7):e69137. doi: 10.1371/journal.pone.0069137 (PMC3712943; doi:10.1371/journal.pone.0069137)

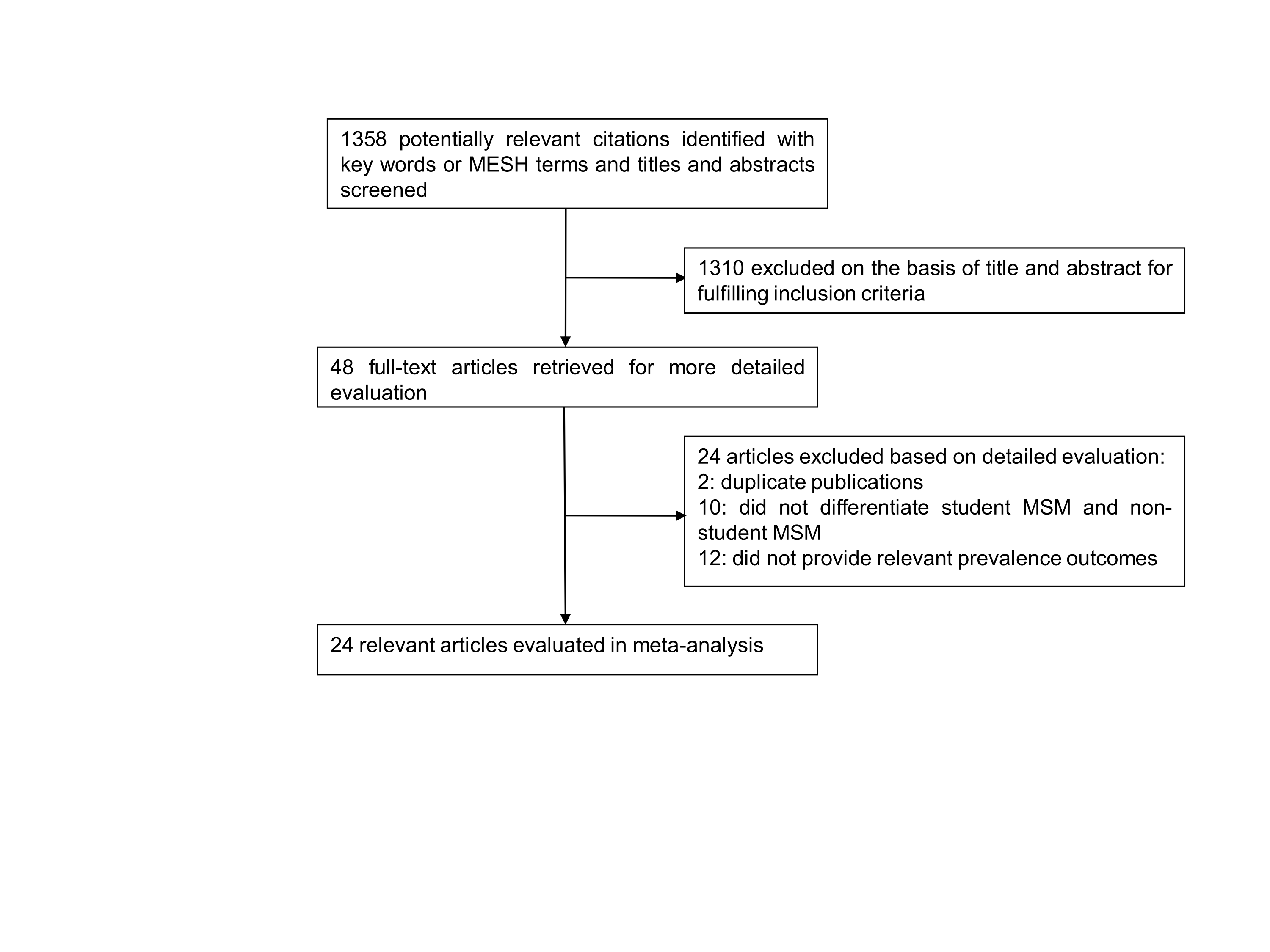

Supplement: Figure S1 — Flow diagram of the study selection process. As shown, our initial searches yielded 1,358 citations from Pubmed, Chinese National Knowledge Infrastructure, Wanfang and Google Scholar databases. After screening titles and abstracts, 48 studies were considered potentially eligible and retrieved in full text. Of these, 24 studies were subsequently excluded because they did not satisfy the inclusion criteria. Thus, 24 fully eligible studies were identified. (TIF) [file pone.0069137.s001.tif]

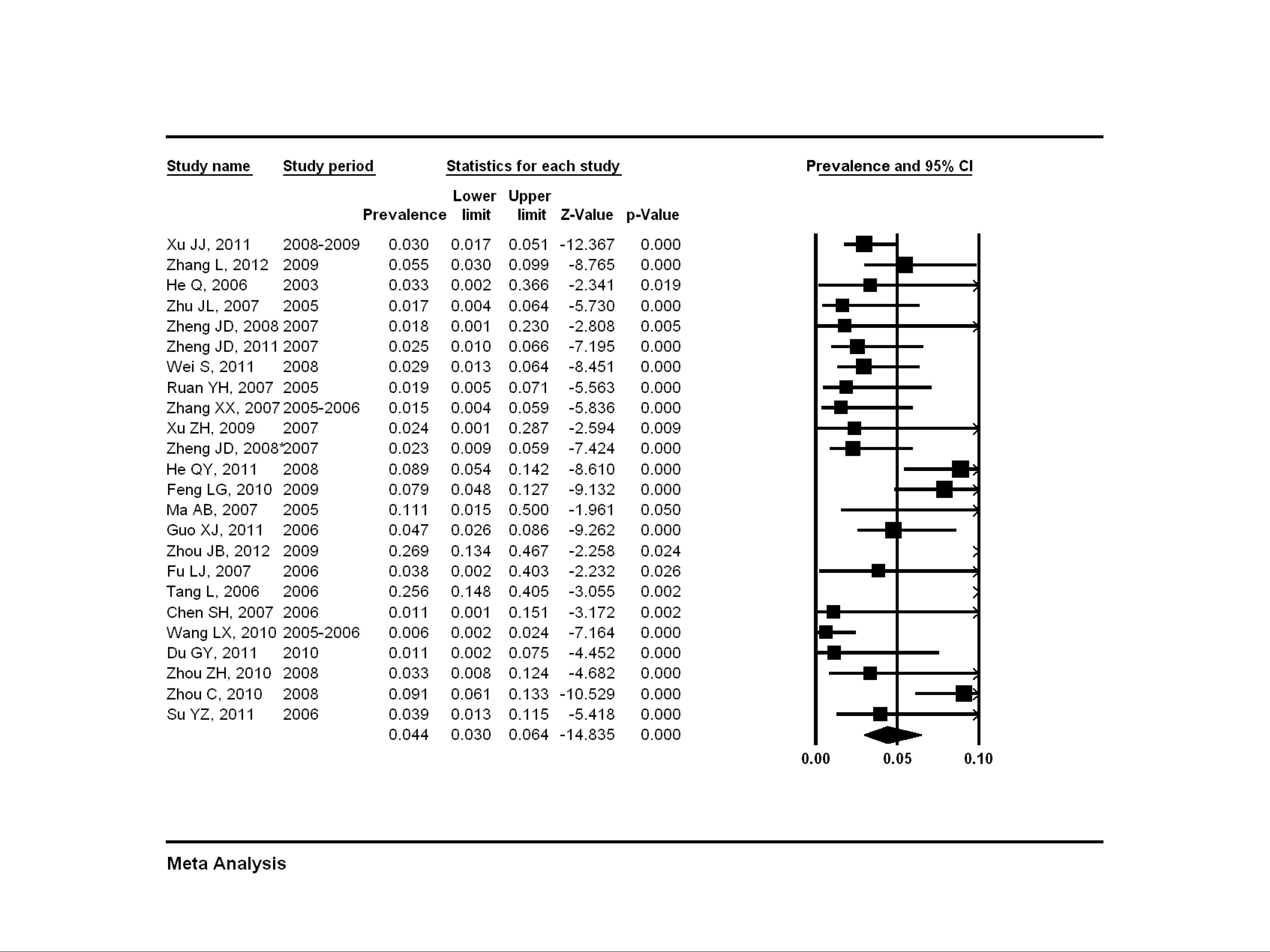

Supplement: Figure S2 — Meta-analysis of the HIV prevalence among student MSM in China. Figure S2 shows unadjusted HIV prevalence estimates (boxes) with 95% confidence limits (bars) for each study selected; pooled prevalence estimates are represented as diamonds in this plot. * This study is different from Zheng JD, 2008. (TIF) [file pone.0069137.s002.tif]

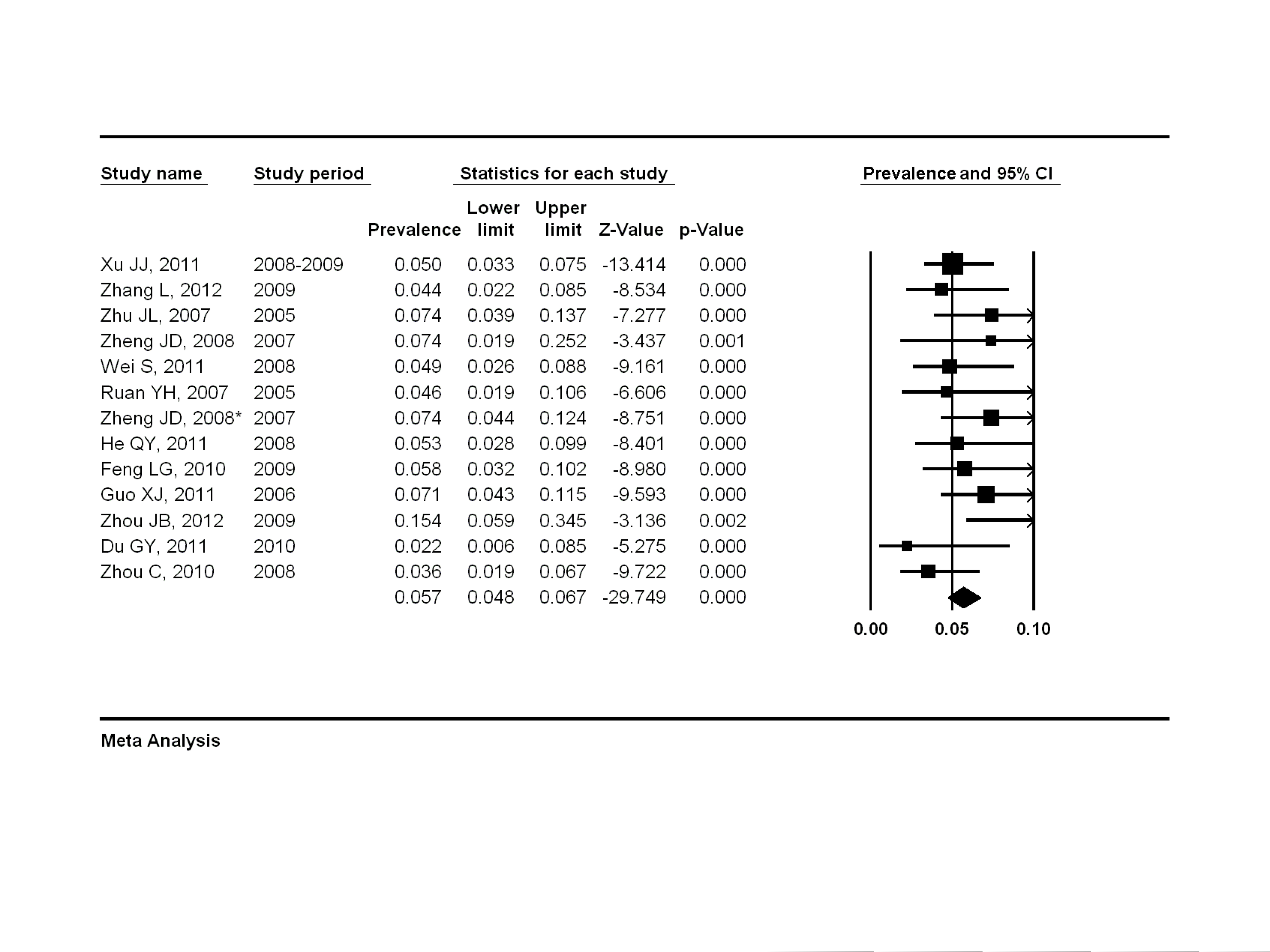

Supplement: Figure S3 — Meta-analysis of syphilis prevalence among student MSM in China. Figure S3 shows unadjusted syphilis prevalence estimates (boxes) with 95% confidence limits (bars) for each study selected; pooled prevalence estimates are represented as diamonds in this plot. * This study is different from Zheng JD, 2008. (TIF) [file pone.0069137.s003.tif]

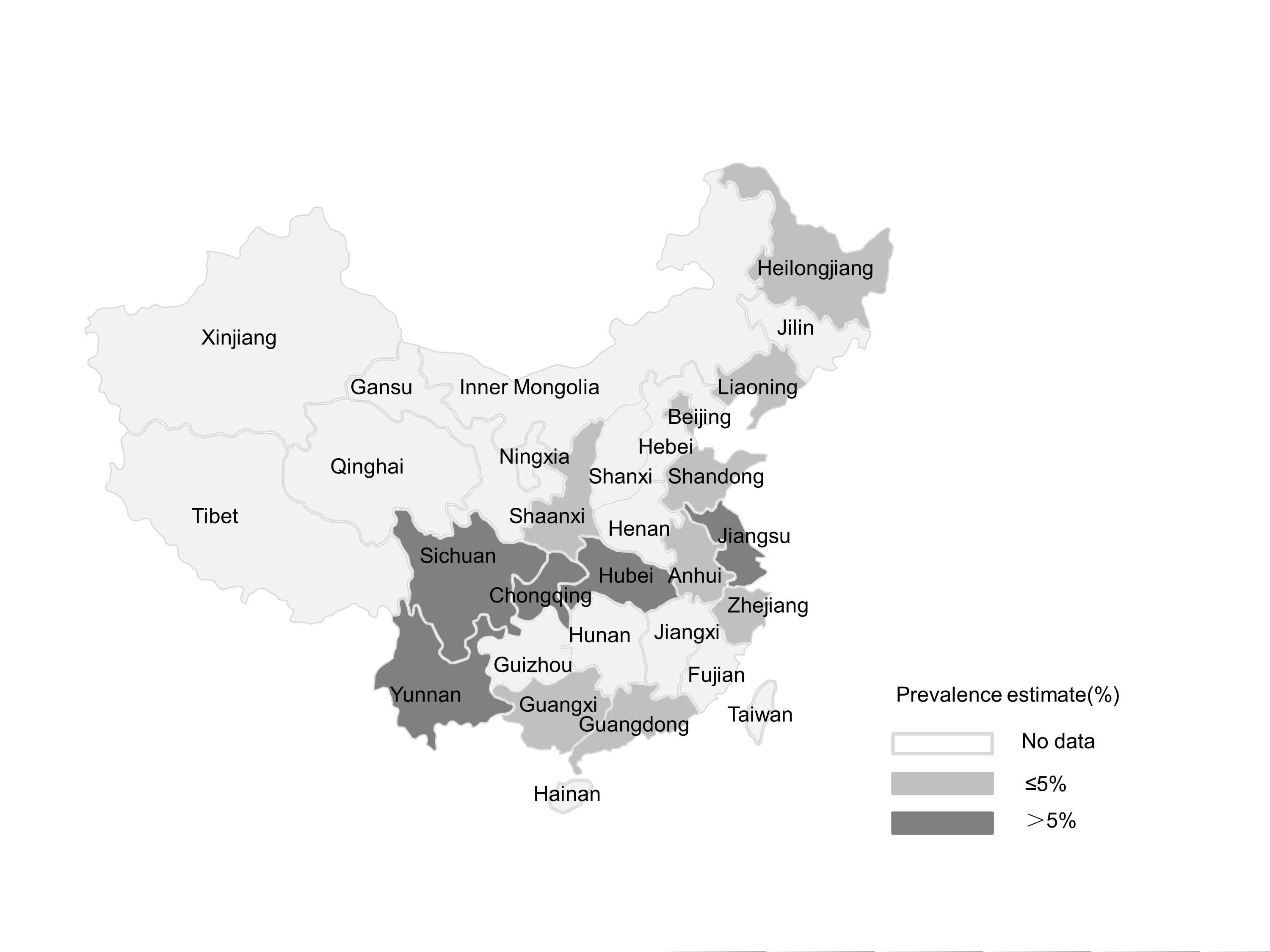

Supplement: Figure S4 — The regional distribution of pooled prevalence of HIV among student MSM in China. (TIF) [file pone.0069137.s004.tif]
